# Plinabulin exerts an anti-proliferative effect via the PI3K/AKT/mTOR signaling pathways in glioblastoma

**DOI:** 10.22038/ijbms.2024.79406.17200

**Published:** 2025

**Authors:** Rouxin Wang, Jing Cheng, Huanqi Zhang, Kaizhi Luo, Rui Wu, Yangling Li, Yuanheng Zhu, Chong Zhang

**Affiliations:** 1 College of Pharmaceutical Sciences, Zhejiang University, Hangzhou 310058, China; 2 School of Medicine, Hangzhou City University, Hangzhou 310015, China; 3 Department of Clinical Pharmacology, Hangzhou First People’s Hospital, Hangzhou 310006, China; 4 Department of Pharmacy, Zhejiang University of Technology, Hangzhou 310027, China; 5 Key Laboratory of Novel Targets and Drug Study for Neural Repair of Zhejiang Province, School of Medicine, Hangzhou City University, Hangzhou 310015, China; 6 Department of Pharmacy, Ningbo No.2 Hospital, Ningbo 315010, China

**Keywords:** AKT, Autophagy, EGFR, mTOR, PI3K, PIK3CG, Plinabulin

## Abstract

**Objective(s):**

Plinabulin, a marine-derived anticancer drug targeting microtubules, exhibits anti-cancer effects on glioblastoma cells. However, its therapeutic potential, specifically for glioblastoma treatment, remains underexplored. This study aims to elucidate the mechanisms by which plinabulin exerts its effects on glioblastoma cells.

**Materials and Methods:**

Using the SRB and colony formation assay to observe the effect of plinabulin on glioblastoma cell viability. Wound healing and transwell migration assay were used to test the effect of plinabulin on glioblastoma cell metastatic potential. Crucial target genes were identified through RNA sequencing and bioinformatics analysis. Protein levels were evaluated in a concentration-dependent manner using western blot analysis.

**Results:**

Plinabulin suppressed glioblastoma cell proliferation by causing cell cycle G2/M phase arrest and inhibited migration. The IC50 values were 22.20 nM in A172 cells and 20.55 nM in T98G cells. Plinabulin reduced AKT and mTOR phosphorylation. Combined with the AKT/mTOR inhibitors LY294002 and rapamycin, plinabulin decreased p-mTOR and EGFR protein levels and increased cleaved-PARP levels. Plinabulin induces autophagy, and using an autophagy inhibitor enhances plinabulin-induced cell apoptosis. This suggests that plinabulin might trigger cytoprotective autophagy in glioblastoma cells. These findings indicate that plinabulin hinders glioblastoma growth and induces protective autophagy via the PI3K/AKT/mTOR pathway. Additionally, plinabulin combined with erlotinib showed greater cytotoxic efficacy than either drug alone in glioblastoma cells *in vitro*.

**Conclusion:**

Our study provides new insights into the efficacy of plinabulin against glioblastoma and highlights the potential clinical utility of combining plinabulin with EGFR inhibitors as a chemotherapy strategy.

## Introduction

Glioblastoma accounts for approximately 49% of malignant brain tumors and is characterized by a median survival of less than two years and a median progression-free survival of about seven months (1). Multimodal treatments, including cytoreductive surgery followed by radiotherapy alongside concomitant and adjuvant temozolomide chemotherapy, have been employed (2). TMZ, a primary chemotherapeutic drug used in glioblastoma, causes substantial DNA damage, whereas enhanced DNA damage repair mainly contributes to TMZ resistance (3). It consistently develops resistance to conventional treatments, resulting in recurrence and heightened mortality (4). Given the poor survival with currently approved treatments for GBM, new therapeutic strategies are urgently needed. Plinabulin (BPI-2358, formerly NPI-2358) is a small non-G-CSF molecule from marine natural products (5) that exhibits the ability to stabilize intracellular microtubule formation *in vitro *(6). Multi-clinical trials have reported it could prevent chemotherapy-induced neutropenia in several solid tumors such as breast (7), prostate (8), or non–small cell lung cancer (9, 10). Therefore, plinabulin is a natural marine product that holds promise for further development. Plinabulin exhibits anticancer activity in multiple myeloma cells, triggering JNK-mediated apoptosis and angiogenesis (11). However, whether plinabulin affects glioblastoma and its molecular mechanism remains largely undiscovered.

Phosphatidylinositol 3-kinase (PI3K)/protein kinase B (AKT)/mammalian target of rapamycin (mTOR) signaling pathway is among the most frequently activated pathways in various human cancers, crucially contributing to the initiation and progression of tumors (12). Alteration of PI3K leads to dysregulation of AKT and then phosphorylates mTOR through a series of regulations (13). Autophagy, an evolutionarily conserved pathway, plays a crucial role in the degradation of excess proteins and organelles (14). The PI3K/AKT/mTOR pathway is a well-established autophagy pathway activated by various receptors and regulators of lysosomal biogenesis and autophagy gene expression. The mTOR activation suppresses autophagy initiation and autophagosome formation (15, 16). Accumulating evidence suggests targeted autophagy may represent an effective therapeutic strategy against glioblastoma (17). Several drugs, such as GANT-61, lactucopicrin, and nanomicellar-curcumin, have been identified as having associations with GBM autophagy (18). Amid therapeutic options that have seen limited success in glioblastoma, modulation of autophagy regulation has become a new avenue of investigation. Our discoveries unveil a previously undisclosed and novel effect of plinabulin in the treatment of glioblastoma. Plinabulin triggers antiproliferative activity via G2/M arrest in glioblastoma cells. Moreover, plinabulin induces autophagy and apoptosis by inhibiting the PI3K/AKT/mTOR pathway. Meanwhile, plinabulin might be a potential sensitizer for EGFR-TKI in glioblastoma treatment. These preclinical studies provide better options in the development of plinabulin as a novel therapy to improve the outcome in glioblastoma patients.

## Materials and Methods

***Drug and reagents***

Plinabulin (714272-27-2) was purchased from Alladin and dissolved in DMSO. Chloroquine phosphate (50-63-5) was purchased from Alladin. 3-MA (S2767), mitomycin C (S8146), LY294002 (S1105), and Baf-A1 (S1413) were purchased from Sellect Chemicals. Rapamycin (R-5000) was purchased from LC Laboratories. Erlotinib hydrochloride (A8234) was purchased from APExBIO.

The antibodies against Cyclin B1 (13667S), E-cadherin (3195S), LC3A/B (12741S), cleaved-PARP (5625S), and phospho-mTOR (5536S) were purchased from Cell Signaling Technology. Antibodies against N-cadherin (22018-1-Ap), Mcl-1 (16225-1-AP), phospho-AKT (66444-1-Ig), EGFR (66455-1-Ig), and GAPDH (60004-1-Ig) were purchased from Proteintech.

***Cell culture***

T98G was procured from Procell Life Science & Technology Co., Ltd (Wuhan, China). A172 cells were procured from the National Collection of Authenticated Cell Cultures. T98G cells were cultured in MEM medium with L(+)-Glutamine, nonessential amino acid, sodium pyruvate, penicillin-streptomycin solution, and 10% fetal bovine serum (FBS). A172 cells were cultured in a DMEM medium with a penicillin-streptomycin solution and 10% FBS (19). All the cells were maintained in a humidified incubator containing 5% CO_2_ at 37 ^°^C.

***Cell viability assay***

Cells (2.5 × 10^3^ cells/well) were plated onto 96-well plates and allowed to adhere overnight. After adherence, the cells were treated with plinabulin and incubated for 24, 48, and 72 hr. Subsequently, cell proliferation was assessed using the sulforhodamine blue (SRB) assay. The cells were fixed with 15% trichloroacetic acid (100 μl per well) for 4 hr, followed by washing with distilled water. After drying in a 37 ^°^C oven, the cells were stained with 0.4% SRB solution for 30 min at room temperature. The dye was removed using 1% acetic acid and dissolved in a Tris-base solution. The cell viability assay was examined at 570 nm with a multiscan spectrum.

***Colony formation assay***

Cells (1.5 × 10^3^ cells/well) were plated onto 6-well culture plates and incubated with plinabulin for 14  days. The medium was replaced every three days. Colonies formed by the remaining cells were stained with a 1% crystal violet solution for 30 min. After washing with distilled water, images of the stained colonies were captured using a microscope.

***Wound healing assay***

T98G and A172 cells (4×10^4^ cells/well), after being treated with plinabulin, were cultured in 24-well plates for 24 hr. To inhibit proliferation, the cells were pre-treated with one μg/ml mitomycin C for 1 hr. Following this, scratches were made in the wells using a sterile 10 µl pipette tip and washed with PBS. Next, the cells were incubated with 500 µl MEM or DMEM medium supplemented with 5% FBS. Cell images were captured using the ImageJ software package at 0 hr and 24 hr.

***Transwell migration assay***

Cells (4 ×10^4^ cells/ml) were seeded in 200 μl serum-free MEM or DMEM medium in the transwell chambers for migration assay. Following this, 600 μl of MEM or DMEM containing 20% FBS was added to the lower compartments of the transwell chambers in 24-well plates. After incubation for 24 hr overnight, cells on the bottom surface of the membrane were fixed and stained with a 1% crystal violet solution for 30 min. The quantification of migrated cells was calculated and recorded using the ImageJ software package.

***Western blot analysis***

Protein lysates were separated by 8-15% SDS-PAGE and then transferred onto PVDF membranes. Following blocking with 5% skim milk to minimize nonspecific binding, the membranes were incubated with primary antibodies overnight at 4 ^°^C. Following this, they were exposed to secondary antibodies for 1 hr at room temperature. Bands were visualized using an imaging system.

***RNA-sequence***

Cells were seeded in 10 cm culture dishes (2×10^6^cells/well), then treated with Plinabulin (50 nM) for 24 hr. Then, cells were washed with cold PBS 3 times, and total RNA was extracted using triazole reagent. The samples were collected and sent for RNA-sequence analysis. Novogene Company (Shanghai, China) conducted RNA-sequence analysis without prior knowledge.

***Bioinformatics analysis***

Using Gene ontology Resource (https://geneontology.org/) to detect the key events regulated by plinabulin (20, 21). Employing SwissTargetPrediction databases (http://swisstargetprediction.ch/) to predict molecular targets of plinabulin (22). Using the GeneCards database (https://www.genecards.org/) to detect genes correlated with glioblastoma (23). Employing UALCAN (http://ualcan.path.uab.edu/analysis.html) database to ascertain the mRNA and protein expression of PIK3CG in glioblastoma and normal brain tissues (24, 25). Utilizing STRING (https://cn.string-db.org/) and Cytoscape (https://cytoscape.org/) to construct a PPI network (26, 27). Utilizing Sangerbox 3.0 (http://sangerbox.com/home.html) to gather overall survival, disease-specific survival, and progression-free interval for glioblastoma patients (28). SynergyFinder (https://synergyfinder.org/#!/) is used to determine synergy scoring via the HSA method (29, 30).

***Statistical analysis***

The data were presented as means±SD. Each experiment was conducted three times to ensure consistency and robustness. Differences between the groups were determined with Student’s *t-test* or one-way ANOVA analysis, and significance was defined as *P<0.05; **P<0.01; ***P<0.001. 

## Results

***Plinabulin inhibits cell viability and induces cell cycle arrest at the G2/M phase in glioblastoma***

**Figure 1:**
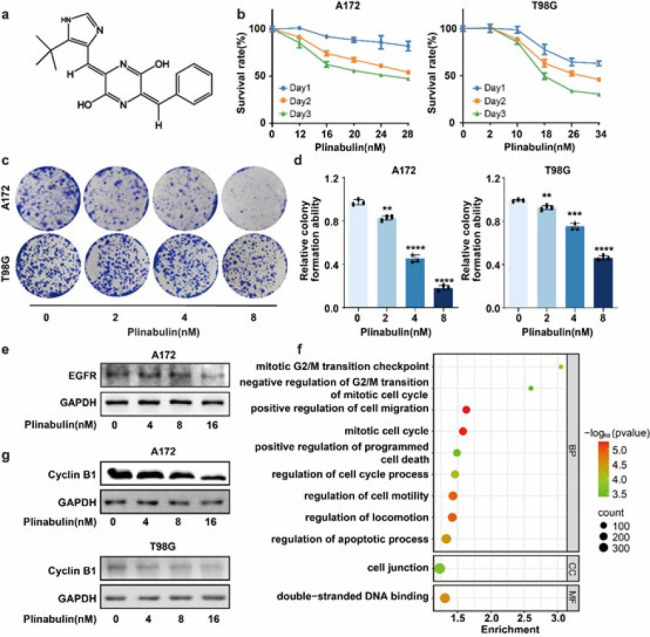
Plinabulin inhibits cell viability and induces cell cycle arrest at the G2/M phase in glioblastoma

Initially, we assessed the impact of plinabulin on the cell proliferation of human glioblastoma cells. The results indicated a time-dependent decrease in cell viability in response to plinabulin. Half-maximal inhibitory concentrations (IC_50_) were 51.99 nM (24 hr), 32.33 nM (48 hr), 22.20 nM (72 hr) in A172, and 50.45 nM (24 hr), 30.92 nM (48 hr), 20.55 nM (72 hr) in T98G (Figure 1b). Meanwhile, plinabulin exposure resulted in the inhibition of colony formation both in A172 and T98G cells (Figure 1c-d). Furthermore, the expression of EGFR in glioblastoma cells was down-regulated by plinabulin treatment (Figure 1e). Collectively, plinabulin effectively inhibits the cell proliferation of glioblastoma cells. RNA-sequence analysis was conducted on glioblastoma to elucidate the molecular mechanism of plinabulin in glioblastoma cells treated with either DMSO or plinabulin. This analysis identified 2664 differentially expressed genes (DEGs) regulated by plinabulin in glioblastoma cells. Functional enrichments of these genes were performed to detect the key events regulated by plinabulin (Figure 1f and Table S1). Plinabulin might be correlated with these events, including mitotic G2/M transition checkpoint, negative regulation of G2/M transition in the mitotic cell cycle, positive regulation of cell migration, mitotic cell cycle, positive regulation of programmed cell death, regulation of cell cycle process, cell motility, locomotion, apoptotic process, cell junction, and double-stranded DNA binding. As anticipated, plinabulin could suppress the expression of cyclin B1 (Figure 1g). These data strongly suggest that plinabulin induces cell cycle arrest at the G2/M phase in glioblastoma cells.

***Plinabulin inhibits the metastatic potential of glioblastoma***

**Figure 2:**
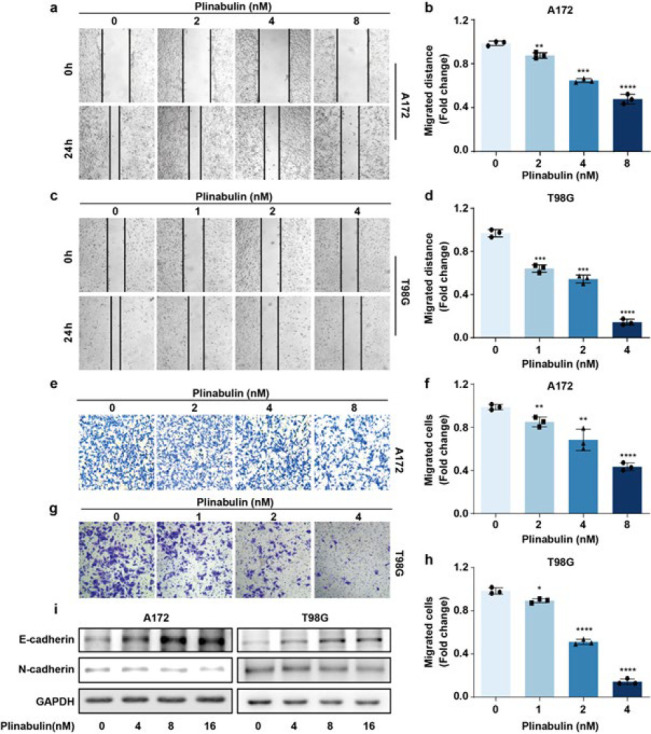
Plinabulin inhibits the metastatic potential of glioblastoma

Given the correlation between plinabulin and cell migration and motility identified in the GO enrichment analysis, we hypothesized that plinabulin could affect the metastatic potential of glioblastoma. Indeed, the wound healing assay revealed a dose-dependent decrease in the migration of glioblastoma cells toward the center of the scratched area (Figure 2a-2d). Meanwhile, the transwell migration assay indicated a significant dose-dependent reduction in the migration of glioblastoma cells induced by plinabulin (Figure 2e-2h). The effect of plinabulin on the regulation of epithelial-mesenchymal transition (EMT) was evaluated by determining the expression of E-cadherin and N-cadherin in glioblastoma cells. Our data showed that plinabulin led to a dose-dependent increase in the expression of E-cadherin and a decrease in the expression of N-cadherin (Figure 2i). Thus, plinabulin suppresses the EMT abilities of glioblastoma cells.

***Plinabulin blocks the PI3K/AKT/mTOR signaling pathway in glioblastoma***

**Figure 3:**
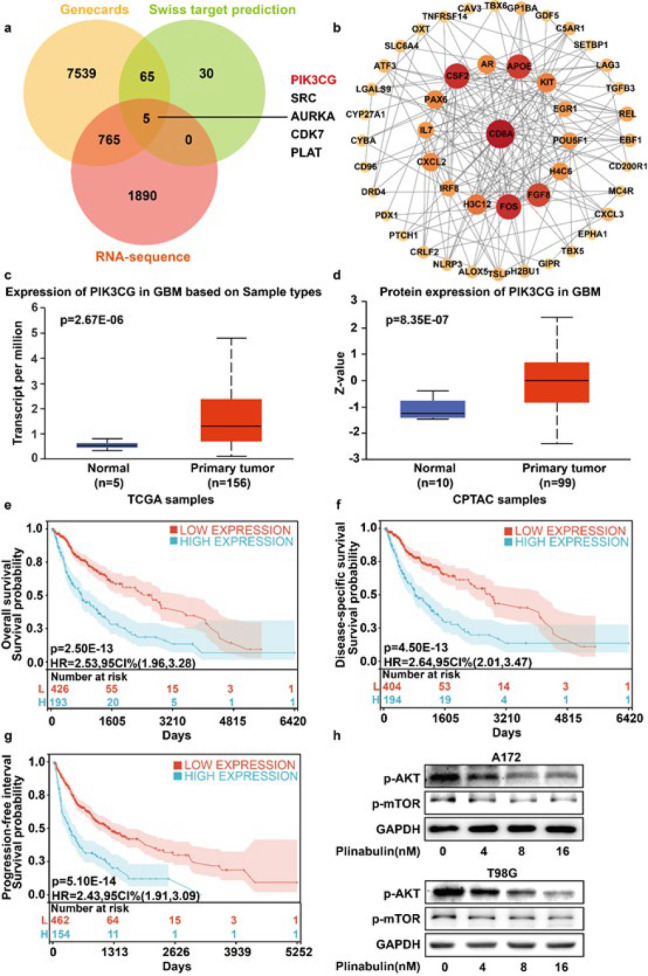
Plinabulin blocks the PI3K/AKT/mTOR signaling pathway in glioblastoma

To better understand the anti-cancer activity of plinabulin in glioblastoma cells, we employed the SwissTargetPrediction databases to predict 100 potential proteins targeted by plinabulin (Table S2). Meanwhile, we also collected 2664 plinabulin-regulated DEGs and 8374 genes correlated with glioblastoma using the GeneCards database (Table S3). To pinpoint the crucial target genes of plinabulin in glioblastoma, a Venn diagram was generated, revealing five overlapping genes among plinabulin-target proteins, plinabulin-regulated DEGs, and glioblastoma co-related genes from GeneCards (Figure 3a). PIK3CG, SRC, AURKA, CDK7, and PLAT might be key molecular targets of plinabulin in glioblastoma. Furthermore, we observed that PIK3CG was the maximum differential expressed gene between the plinabulin-treated group and the control group in glioblastoma. The protein-protein interaction (PPI) network modulated by plinabulin in glioblastoma was constructed using Cytoscape and is depicted in Figure 3b (Table S4). To ascertain the expression of PIK3CG in glioblastoma and normal brain tissues, we examined the mRNA expression of PIK3CG in 5 normal brain tissues and 156 glioblastoma samples from the UALCAN database (Figure 3c). The findings demonstrated that glioblastoma samples had considerably greater mRNA levels of PIK3CG than normal brain tissues (*P*=2.67E-6). Similarly, the protein level of PIK3CG was also elevated in glioblastoma compared to normal brain tissues based on the UALCAN database (normal: n=10 vs glioblastoma: n=99, *P*=8.35E-7, Figure 3d). Meanwhile, the overexpression of PIK3CG is closely correlated with poor overall survival (Figure 3e), disease-specific survival (Figure 3f), and progression-free interval (Figure 3g) of glioblastoma patients. Meanwhile, p-mTOR and p-AKT expression levels exhibited a significant and dose-dependent reduction upon treatment with plinabulin (0, 4, 8, and 16 nM)(Figure 3h).

***Plinabulin regulates the PI3K/AKT/mTOR pathway***

**Figure 4:**
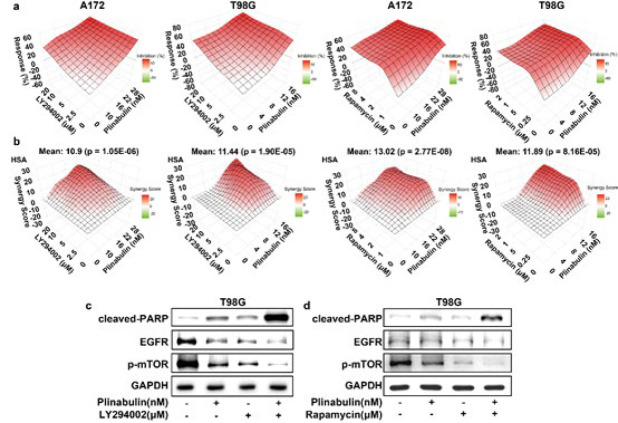
Plinabulin regulates PI3K/AKT/mTOR pathway

To provide additional validation of plinabulin’s ability to modulate the PI3K/AKT/mTOR signaling pathway, we subjected cells to pre-treatment with the PI3K inhibitor (LY294002) and mTOR inhibitor (rapamycin) prior to plinabulin administration. Our findings revealed that prior exposure to LY294002 or rapamycin further significantly intensified cell viability in plinabulin-treated cells (Figure 4a). Besides, SynergyFinder was used to determine synergy scoring via HSA method, the results indicated a pronounced synergistic cytotoxic effect when LY294002/rapamycin were combined with plinabulin in two glioblastoma cells (Figure 4b). Simultaneous pretreatment with LY294002 and rapamycin notably augmented the reduction in p-mTOR and EGFR levels induced by plinabulin. The combined treatment of LY294002 or rapamycin with plinabulin significantly increased the expression of cleaved-PARP compared to either agent alone (Figure 4c-d). These data suggest that plinabulin induces apoptosis by inhibiting the PI3K/AKT/mTOR pathway. The cumulative findings strongly indicate that plinabulin manifests its cytotoxic effect in glioblastoma cells through modulation of the PI3K/AKT/mTOR signaling pathway.

***Plinabulin triggers cytoprotective autophagy in glioblastoma***

Plinabulin demonstrated a dose-dependent increase in the conversion from LC3-I to LC3-II in A172 and T98G cell (Figure S1a). To determine whether autophagy induced by plinabulin has a protective or detrimental impact on glioblastoma cells, we utilized two well-established autophagy inhibitors, Baf-A1 and CQ. Pretreatment of Baf-A1 or CQ significantly reduced cell viability in plinabulin-treated cells with highly synergistic cytotoxic effect, which suggested that plinabulin induces protective autophagy (Figure S1b-c). Further, plinabulin plus CQ significantly enhanced apoptosis compared with either single agent (Figure S1d). 

We utilized 3-MA, an inhibitor of PI3K and autophagy, to further explore its impact on glioblastoma cells when combined with plinabulin treatment. Compared with either single agent, the combination of plinabulin and 3-MA markedly suppressed the expression of p‐mTOR and EGFR and enhanced apoptosis (Figure S1e). 

***Plinabulin overcomes resistance to EGFR inhibitors in glioblastoma***

**Figure 5:**
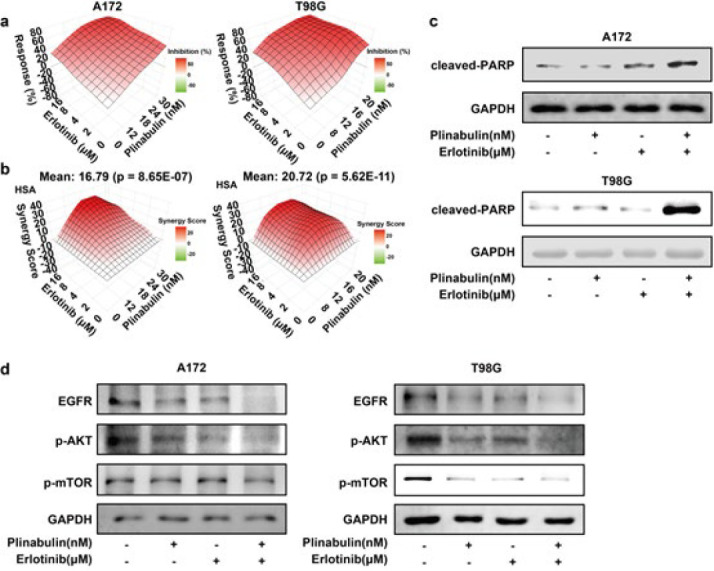
Plinabulin overcomes resistance to EGFR inhibitors in glioblastoma

The effect of plinabulin plus erlotinib was then evaluated on A172 and T98G cells, and the viability of glioblastoma cells was detected after the treatment of plinabulin and/or erlotinib. The highly synergistic cytotoxic effect of plinabulin plus erlotinib was observed in glioblastoma cells using SRB assay (Figure 5a-b). In comparison to the groups treated with either single agent alone, the combination of plinabulin and erlotinib significantly elevated the expression of cleaved-PARP (Figure 5c). These data suggested that plinabulin enhanced the cytotoxic effect of erlotinib via suppressing cell proliferation and inducing apoptosis. On the other hand, combination treatment of plinabulin with erlotinib demonstrated a significant suppression of EGFR, p-AKT, and p-mTOR protein expression, in comparison to either agent alone (Figure 5d). Collectively, these findings showed plinabulin sensitized the cytotoxic effect of erlotinib in glioblastoma cells.

## Discussion

Plinabulin, a marine natural product, is gaining increasing significance due to its potential in cancer therapeutics (31). Recently, plinabulin has shown antitumor activity against different types of tumors, encompassing colorectal, prostate, breast, non-small cell lung cancer, multiple myeloma, and leukemia (32). Nevertheless, the effect of plinabulin on glioblastoma is currently unclear. Our study firstly illustrated that plinabulin inhibited cell proliferation and migrative ability in a dose-dependent manner on glioblastoma cells. Several studies have indicated that plinabulin inhibits cell-cycle progression, specifically during the M phase, particularly around the prometaphase and metaphase (33, 34). We found, as expected, that plinabulin arrested the cell cycle in G2/M in glioblastoma cells. Considering that the M phase is the most radiosensitive stage of the cell cycle (35), synchronizing in the early M phase would represent an optimal radio sensitization strategy.

Multiple researchers have confirmed that activation of PI3K signaling can contribute to tumorigenesis and is a hallmark of human cancer (36). The PI3K/AKT/mTOR (PAM) signaling pathway constitutes a pivotal transduction network that facilitates cell survival, growth, and progression through the cell cycle. Furthermore, the over-activity of PAM promotes EMT and metastasis by significantly influencing cell migration (37). The type 1B PI3K p110γ (PIK3CG), a type of subunit of PI3K, is activated by interaction with G-protein-coupled receptors. The inhibition of the PIK3CG gene is pivotal in dampening the PAM signaling pathway, thereby hindering tumorigenesis and the advancement of colorectal cancers (38). Our study first demonstrated that plinabulin might be a potential inhibitor of PIK3CG, and served as an anti-glioblastoma agent. Plinabulin inhibits the activation of AKT and mTOR proteins, as evidenced by reduced phosphorylation. The cytotoxic effect of plinabulin is significantly enhanced when combined with LY294002 (PI3K agonist) and rapamycin (mTOR agonist), confirming its role in promoting glioblastoma cell death by inhibiting the PAM pathway. Despite these discoveries, the intracranially orthotopic transplantation model or subcutaneous xenograft model could be used for further elucidation of plinabulin’s therapeutic potential on GBM in* vivo*.

Around 50% of glioblastomas exhibit activating mutations, amplification, or overexpression of EGFR, leading to the activation of downstream PI3K/AKT/mTOR pathways. Due to its pivotal role in glioblastoma progression, EGFR has emerged as a compelling therapeutic target (39). Mutational activation of the EGFR and downstream PI3K signaling components have been recognized as novel mechanisms of resistance to TKIs (40-42). Eganelisib, a small molecular inhibitor targeting PIK3CG, demonstrated notable therapeutic efficiency in gefitinib-resistant cells (43). More importantly, the plinabulin and erlotinib combination exerted a more cytotoxic effect compared with single-agent alone, indicating that plinabulin might be an effective sensitizer for erlotinib in glioblastoma treatment. Furthermore, plinabulin plus erlotinib decreased the expression of EGFR, p-AKT, and p-mTOR, resulting in cell growth reduction.

**PIK3CG inhibitors, including duvelisib and IPI-549, exhibit promise in leukemia and breast cancer, respectively (44, 45). However, their application in glioblastoma is unexplored. In this study, PIK3CG was significantly down-regulated in plinabulin-treated group compared with non-treated group, and we first demonstrated that plinabulin was regarded as a novel PIK3CG inhibitor and might be an efficacious anti-cancer agent in glioblastoma treatment. Recent studies highlight the pivotal role of PIK3CG, an emerging immune checkpoint, particularly involving tumor-associated macrophages (TAMs) (46). Plinabulin, identified for its immunomodulatory effects, induces M1 polarization and enhances anti-tumoral functions in TAMs (47), fosters dendritic cell maturation, and promotes antitumor T cell responses (48). Thus, we hypothesized that plinabulin might be a potential immunomodulatory agent in glioblastoma immunotherapy, but this might need further investigation. Clinical trials explore plinabulin’s synergy with pembrolizumab and docetaxel for non-small cell lung cancer (49). Thus, we also assumed that the combination treatment of plinabulin with pembrolizumab and docetaxel might also be effective in glioblastoma treatment, and plinabulin is a potential candidate for combination therapy to overcome immune checkpoint inhibitor resistance in glioblastoma treatment.**

a, Molecular structure of plinabulin. b, Glioblastoma cells were treated with DMSO or plinabulin at the indicated concentrations for 1-3 days, and SRB assay was conducted to assess glioblastoma cell proliferation. c-d, Glioblastoma cells were incubated with DMSO or plinabulin for 14 days, and the colony count of glioblastoma cells was determined. e, Glioblastoma cells were treated with DMSO or various concentrations (4, 8, 16 nM) of plinabulin for 24 hr, and EGFR expression levels were analyzed by western blot. f, 2664 DEGs regulated by plinabulin underwent functional enrichment analysis using the Gene Ontology Resource database. g, Glioblastoma cells were treated with DMSO or various concentrations (4, 8, 16 nM) of plinabulin for 24 hr, and cyclin B1 expression was analyzed by Western blot

a-d, Glioblastoma cells were exposed to DMSO or plinabulin for 24 hr, and the migratory capacities of cancer cells were assessed using the wound healing assay. e-h, Glioblastoma cells were exposed to DMSO or plinabulin for 24 hr, and the migratory capacities of cancer cells were evaluated through the transwell assay. i, Glioblastoma cells were treated with DMSO or various concentrations (4, 8, 16 nM) of plinabulin for 24 hr, and the levels of E-cadherin and N-cadherin were analyzed by Western blot

a, Identification of DEGs was conducted via RNA-sequence, the plinabulin molecular targets were gathered from SwissTarget Prediction, and genes correlated with glioblastoma were sourced from GeneCards. b, A significance threshold for selecting DEGs regulated by plinabulin was set at P<0.05, with a Log2fold-change above 2.5 or below -2.5. The construction of PPI network utilized STRING and under went additional analysis in Cytoscape. c, The data was collected from UALCAN. Gene:PIK3CG; TCGA dataset: Glioblastoma multiforme. d, The result was acquired from UALCAN. Gene: PIK3CG; CPTAC dataset: Glioblastoma multiforme. e, Overall survival data for glioblastoma patients were gathered from Sangerbox 3.0. Gene:PIK3CG; Cancer Type: glioblastoma; Survival Measure: overall survival. f, The disease-specific survival for glioblastoma patients was obtained from Sangerbox 3.0. Gene: PIK3CG; Cancer Type: glioblastoma; Survival Measure: disease-specific survival. g, The progression-free interval of glioblastoma patients was collected from Sangerbox 3.0. Gene: PIK3CG; Cancer Type: glioblastoma; Survival Measure:progression-free interval. h, The glioblastoma cells were exposed to DMSO or various concentrations (4, 8, 16 nM) of plinabulin for 24 hr, and the expression levels of p-mTOR and p-AKT were analyzed through Western blot

a-b, Glioblastoma cells underwent a pretreatment phase with LY294002/rapamycin lasting 1 hr, followed by treatment with DMSO or plinabulin at the indicated concentrations for 72 hr, and the assessment of synergy scoring was conducted using the online SynergyFinder software. c, Glioblastoma cells underwent a pretreatment phase with 20 μM LY294002, followed by a 12 nM plinabulin treatment, while the control group was treated with DMSO. The expressions of p-mTOR and EGFR were assessed using western blot lasting 24 hr, the expression of cleaved-PARP was evaluated through western blot analysis, extending the observation period to 48 hr. d, Glioblastoma cells underwent a pretreatment phase with 16 μM rapamycin, followed by a 12 nM plinabulin treatment, while the control group was treated with DMSO. The expression levels of p-mTOR and EGFR were assessed using Western blot analysis, with a duration of 24 hr, the expression of cleaved-PARP was evaluated through western blot for 48 hr

a-b, glioblastoma cells were treated with plinabulin and/or erlotinib at specified concentrations for a duration of 72 hr, while the control group was treated with DMSO, and the assessment of synergy scoring was conducted using the online SynergyFinder software. c, A172 cells were incubated with 24 nM plinabulin and/or 24 μM erlotinib, T98G cells were incubated with 16 nM plinabulin and/or 16 μM erlotinib for 48 hr, while the control group was treated with DMSO. The expression of cleaved-PARP was assessed using western blot. d, A172 cells were incubated with 24 nM plinabulin and/or 24 μM erlotinib, T98G cells were incubated with 16 nM plinabulin and/or 16 μM erlotinib for 24 hr, while the control group was treated with DMSO. The levels of p-mTOR, EGFR, p-AKT were evaluated using Western blot

## Conclusion

In summary, our study comprehensively demonstrated that plinabulin, a diketopiperazine microtubulin inhibitor, possesses antitumor characteristics in glioblastoma cells. Plinabulin effectively inhibited the proliferation of glioblastoma cells and induced autophagy by targeting the PI3K/AKT/mTOR pathway. Additionally, plinabulin enhanced the anti-glioblastoma activity of erlotinib. These encouraging findings lay a foundation for potential clinical applications, suggesting that plinabulin, either alone or in combination with erlotinib, might serve as a viable strategy in glioblastoma treatment.

## Data Availability

The data presented in the study are included in the article and additional material.

## Declaration

We acknowledge the use of ChatGPT 3.5 to enhance language quality and check for grammatical errors. After using this tool, we reviewed and edited the content as needed and take full responsibility for the content of the publication.
